# Does periodontitis represent a risk factor for rheumatoid arthritis? A systematic review and meta-analysis

**DOI:** 10.1177/1759720X19858514

**Published:** 2019-07-09

**Authors:** Railson de Oliveira Ferreira, Raíra de Brito Silva, Marcela Baraúna Magno, Anna Paula Costa Ponte Sousa Carvalho Almeida, Nathália Carolina Fernandes Fagundes, Lucianne Cople Maia, Rafael Rodrigues Lima

**Affiliations:** Laboratory of Functional and Structural Biology, Universidade Federal do Pará, Belém, Brazil; Laboratory of Functional and Structural Biology, Universidade Federal do Pará, Belém, Brazil; Department of Pediatric Dentistry and Orthodontics, Universidade Federal do Rio de Janeiro, Rio de Janeiro, Brazil; Laboratory of Functional and Structural Biology, Universidade Federal do Pará, Belém, Brazil; Laboratory of Functional and Structural Biology, Universidade Federal do Pará, Belém, Brazil; Faculty of Medicine and Dentistry, University of Alberta, Edmonton, Canada; Department of Pediatric Dentistry and Orthodontics, Universidade Federal do Rio de Janeiro, Rio de Janeiro, Brazil; Laboratory of Functional and Structural Biology, Institute of Biological Sciences, Federal University of Pará, Rua Augusto Corrêa 1, Guamá, Belém, PA 66075-900, Brazil

**Keywords:** periodontitis, rheumatoid arthritis, systematic review

## Abstract

Periodontitis is an inflammatory disease of dental supporting tissues (gingiva, periodontal ligament, and bone) and it has been suggested as a possible etiology for rheumatoid arthritis (RA). In this systematic review, we aim to verify if periodontitis represents a risk factor for RA. Electronic databases were consulted until March 2018 considering eligibility criteria focusing on: (P, participants) adults; (E, exposure) with periodontitis; (C, comparison) without periodontitis; and (O, outcome) development of RA. Quality assessment of studies and risk-of-bias evaluation were also performed. To undertake a quantitative analysis, the number of persons with RA and a total number of participants for the case group (with periodontitis) and control group (without periodontitis) were used to calculate the odds ratio (OR) with a 95% confidence interval (CI). A total of 3888 articles were identified, and nine studies were considered eligible. Seven of 9 articles suggested an association among diseases by the common pro-inflammatory profiles. The pooled analysis of 3 articles showed a higher RA prevalence for persons with periodontitis (*n* = 1177) than controls (*n* = 254) (OR 1.97; CI 1.68–2.31; *p* < 0.00001). However, considerable heterogeneity among studies was verified (I^2^ = 96%, *p* < 0.00001). Periodontitis may represent a risk factor for RA by heredity, bacterial infection, and the pro-inflammatory profile shared between both diseases. Although most of the elective studies report an association between periodontitis and RA, the quantitative analysis showed a high heterogeneity, leading to the need for further studies.

## Introduction

Among the inflammatory diseases, periodontitis is one of the most common oral conditions with an inflammatory profile.^1,2^ This disease is an inflammatory impairment that may mildly affect gingiva, resulting in bleeding, halitosis, and edema, or result in severe damage of dental supporting tissues, promoting damage of the gingiva, periodontal ligament, loss of attachment of alveolar bone and tooth loss.^3,4^

The pathogenesis of periodontitis is a result of complex interactions between the periodontal pathogens and immune response.^5^ Some studies have reported the activity of periodontal pathogens and the presence of inflammatory cytokines [interleukin-1 beta (IL-1β), tumor necrosis factor alpha (TNF-α), and others] in systemic inflammatory diseases.^6^ Cardiovascular diseases, such as atherosclerosis, showed associations with periodontitis through inflammatory markers; immune markers also presented in rheumatoid arthritis (RA),^6^ especially C-reactive protein and IL-1β.

RA is an inflammatory disease of joints involving an autoimmune attack of periarticular tissues, which may compromise synovial fluids, joints cartilage, and bone integrity.^7^ The etiology of RA remains unclear, but the activity of periodontal pathogens has been related to the production of RA autoantibodies.^8^ Citrullination of neutrophils and joint tissue proteins performed by *Porphyromonas gingivalis and Aggregatibacter actinomycetemcomitans* is possibly responsible for triggering autoimmune responses and autoantibody production.^5,8^ This way, the inflammatory pattern of both diseases may possibly trigger events that establish the RA disease in periodontitis patients.^9^

In this systematic review and meta-analysis, we aimed to verify whether periodontitis represents a risk factor for the development of RA.

### Material and methods

#### Protocol and registration

This systematic review was registered at PROSPERO under the code CRD42018085004. This study was conducted following the Preferred Reporting Items for Systematic Review and Meta-Analysis guidelines (Supplementary Table 1),^10^ adapted by Penoni *et al*.^11^ and Almeida *et al*.^12^

## Eligibility criteria, search strategy and data extraction

The PECO strategy was used in this systematic review. Observational studies in humans (P, participants) presenting with periodontitis (E, exposure) and the absence of periodontitis (C, comparison), in which the primary outcome (O) was the development of RA in this population, were considered eligible. The null hypothesis of the study was ‘there is no association between periodontitis and the development of RA.’

The searches were conducted in the following electronic databases, without language restriction, until March 2018: PubMed, Scopus, Web of Science, The Cochrane Library, and LILACS. The gray literature was also searched through OpenGrey and Google Scholar. All publications presented in the databases and gray literature contained a combination of controlled predefined Medical Subjects Headings (MeSH) and free terms relating to periodontitis and RA. Boolean operators (or, and) were used to combine searches (Supplementary Table 2).

All relevant citations were saved in a bibliographic reference manager (Endnote x7 version, Thomson Reuters, Philadelphia, PA, USA). Duplicated results were considered only once. Titles and abstracts were analyzed according to inclusion and exclusion criteria. Additional citations were sought from the analysis of the reference list of all articles previously selected. The selection process was conducted by two examiners (ROF and NCFF) and checked by a third examiner (RRL), in case of disagreements.

After the duplicate removal, opinion articles, technical articles, guides, and animal studies were excluded. The titles and abstracts that did not adhere to the established eligibility criteria were excluded. The resulting articles were evaluated and judged by their full text.

## Data extraction

The extraction of data was conducted from the selected articles. A table was used to report year of publication, study design, participant characteristics (source and sample size), age, periodontitis assessment, RA assessment, statistical analysis, and results.

In case of absence of information that makes data extraction or risk-of-bias evaluation impracticable, we attempted to contact the authors by email. The contact consisted of sending a weekly email, for up to 5 consecutive weeks.

## Quality assessment and risk of bias

For assessment of methodologic quality and the risk of bias, the Fowkes and Fulton checklist,^13^ adapted by Almeida *et al*.^12^ was applied. The checklist has domains that assess study designs and study samples, control group characteristics, quality of measurements and results, completeness, and distortion influences.

To provide valid information and feasibility of methods, the checklist was standardized by the examiners. The sign (++) was applied when the analyzed criteria had a major problem, and the sign (+) was used when the research had criteria with a minor problem. The number ‘zero’ (0) was applied when the analyzed criteria had no problems, and the sign NA was marked when the analysis of the topic was not applicable. The criteria used for quality assessment are listed in Table 1. After a detailed analysis of methods and outcomes, questions of the studies were posed to verify bias, considering: the occurrence of biased results, confusing distortions, and results occurring by chance. The summary questions were: ‘Are the results biased?’; ‘Are confusing factors present in the results?’; and ‘Is there a possibility that the results occurred by chance?’. We attributed the answers ‘Yes’ and ‘No’. If an article received ‘No’ to the three questions, it was considered having a low risk of bias.

**Table 1.** Quality assessment and risk of bias according to Fowkes and Fulton.^13^

Guideline	Checklist	Description
Study design appropriate to objectives?	Objective common designPrevalence cross-sectionalPrognosis cohortTreatment-controlled trialCause cohort, case control, cross-sectional	The type of study was marked in the appropriate type of study; if the type of study was appropriate according to the study design was marked as ‘0’ and as ‘++’ if it was not appropriate
Study sample representative?	Source of sampleSampling methodSample sizeEntry criteria/exclusionNonrespondents	The domain was considered (0) in cases of detailed origin, (+) to a specified origin of only one group, and (++) in cases of absence of specification of the origin of the groupsThe item was assigned (0) for a full description of sampling method, (+) for poor or no description of sample method, with no problem in matching between groups and (++) for poor or no description of sample method, interfering in the matching of the groupsA minor problem (+) was considered when the sample was not representative or did not report a sample calculation; to a major problem, (++) was considered when no sample calculation was provided and the number of participants was less than 50 participants; (0) was considered in the absence of the above factorsA minor problem (+) was assigned when the control and case group reported current use of antibiotics or anti-inflammatories, diabetes, smoking, or pregnancy, and in the case of presence of more than two previously mentioned items, it was considered a major problem (++)(0) was assigned when there was no refusal to participate in the study, (+) was assigned when there was a refusal, but did not compromise the sample, and (++) when there were refusal and impairment of the sample size
Control group acceptable?	Definition of controlsSource of controlsMatching/randomizationComparable characteristics	(0) was assigned when all characteristics of a control group were described; (+) when any information was considered originating from the control group, the selection criteria, or a different origin between case and control groups; and (++) when two or more items were described in previously mentioned items(0) was assigned when the control group was referred; (+) when the origin of groups was different, but with reasons; and (++) when the groups present different origins without explanationIn this item, (0) was assigned to cases of randomized/matched groups; (+) to cases of no description of randomization, but with the matching of groups; and (++) to no description of randomization or matching(0) was assigned to matched groups or not matched by the impossibility of being subsequently adjusted and (++) the presence of unpaired variables that were not paired or adjusted
Quality of measurements and outcomes?	ValidityReproducibilityBlindnessQuality control	(0) was assigned when the evaluation method applied was appropriate; (+) when using a single method, but with appropriate sensitivity with good specificity; (++) when using a single method, without an adequate specificity or good sensitivity(0) was assigned to whether the evaluation methods were well described; (+) when lacking description of any step of the method, for example, the identification of patients from the groups studied in laboratory samples, evaluations at different times or application of different methods between groups of specific pathology; (++) when two or more of the previous items are presentWhen the condition of the study participants was considered ‘blind,’ (0) was assigned; in cases of ‘not blind,’ (++) was assignedIt was considered a problem when examiners were not submitted to a standard error analysis (Kappa error analysis); when unqualified students were assessed without supervision by a qualified dentist; when analysis of periodontitis was only radiographic or depth of periodontal pockets only was used; evaluation of less than three dental sites or no mention how many faces were evaluated; two of these problems were identified, it was considered a minor problem (+), and major problem (++) if more than two of these characteristics were described
Completeness	ComplianceDropoutsDeathsMissing data	(0) was assigned for a sample size that remains the same from beginning to end or decreases without compromising the power of the test; (+) for differences in sample size at the end of the study, compromising the power of the test, but with reasons and adjusts; (++) for difference in sample size at the end of the study, compromising the power of the test, without explanationThe (0) was scored when there is no loss during the study, (+) when there was a withdrawal involving the inclusion criteria, such as age, sex, and (++) when there was withdrawal and it compromised more than one criterionThis item was scored as not applicable (NA), using the PECO strategyIn this item, (0) was assigned to cases of randomized/matched groups; (+) to cases of no description of randomization, but with the matching of groups; and (++) to no description of randomization or matching
Distorting influences?	Extraneous treatmentsContaminationChanges over timeConfounding factorsDistortion reduced by analysis	In this item, (0) was considered when there were no external influences; (+) when there were external influences, but did not interfere with the results; (++) when there were external influences and they did interfere with the resultsThis item was scored as NA, using the PECO strategyIn this item, (0) was assigned to data collected in the same period; (+) to data collected from the control and study groups at different times that might cause distortions; (++) when the previous item was associated with data from studies already publishedA problem was considered in the case of men and women under the age of 45, being menopausal, being a smoker, being diabetic and obese women. A ‘minor’ (+) problem was assigned when one or two of these characteristics were present and a ‘larger’ (++) problem if there were three or moreIn this item, (0) was considered when it cites the adjustments of the covariates that present distortions; (+) when the article reports adjustment, but does not specify the criteria; (++) when distortion was identified, without adjustment
Summary questions	Bias: are the results erroneously biased in a certain direction?Confounding: are there any serious confusing or other distorting influences?Chance: is it likely that the results occurred by chance?	‘Yes’ or ‘No’ answers were assigned for each question. If the answer is ‘No’ to the three questions, the article is considered reliable, with a low risk of bias

## Quantitative analysis

The Review Manager software, version 5.3 (The Cochrane Collaboration, Copenhagen, Denmark) was used in the meta-analysis to evaluate the association between periodontitis and the risk of developing RA. The number of participants with RA, the total number of participants for the case group (with periodontitis) and control group (without periodontitis) were used to calculate the odds ratio (OR) with 95% confidence interval (CI).

A fixed-effects model was applied, and heterogeneity was tested using the *I*^2^ index. Sensitivity analyses were conducted to estimate and verify the influence of studies, one by one, on the pooled result.^14^ Publication bias was not quantitatively evaluated by the Egger test or funnel plot, as there were not enough studies to be grouped in a funnel plot.^15^

The original authors were contacted when further results were required. If, after contact attempts, there was no response from the authors, the study was not included in the meta-analysis.

## Results

### Study selection and characteristics

A total of 3888 articles were identified from the searches and 793 articles were excluded because of duplication. Titles and abstracts of 3095 potentially eligible manuscripts were verified following the entry criteria resulting from the exclusion of 3079 articles, and 14 articles were selected for full-text reading.

After this step, four studies were excluded due to their evaluation of established cases of RA^8,16–18^ and due to the inclusion of patients with symptoms of arthralgia.^19^ The reasons for exclusion and citations are presented on Supplementary Table 3.

**Figure 1.**
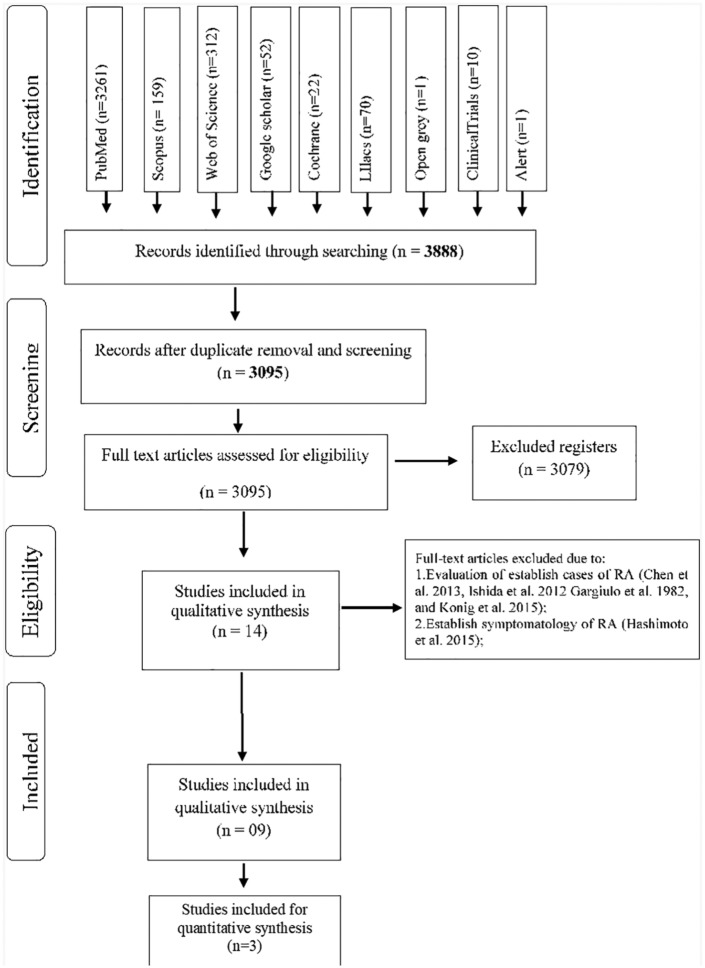
Flow diagram of databases searched according to PRISMA guidelines. PRISMA, Preferred Reporting Items for Systematic Review and Meta-Analysis.

Nine articles were included in this review and their selection process is shown in Figure 1.

## Results of individual studies

Among the nine articles included, two articles present a cohort design,^20,21^ while seven were cross-sectional studies^22–28^ (Table 2). The association between the periodontitis and development of RA were cited in seven^20,22–27^ of nine articles. The absence of association reported was discussed by the authors and it was agreed the reasons were related to the method of periodontitis evaluation.^21,28^ Dichotomous variables (yes or no/presence or absence) as tooth loss and periodontal surgery were associated with studies with higher sample sizes.^20,27^ The other seven articles reported relationships between periodontitis and RA, which were grouped by:

**Table 2.** Summary of characteristics and results of the included studies.

Authors/study design	Participants	Age	Periodontitis diagnosis		Arthritis diagnosis		Statistical analysis	Results	Outcomes
			Clinical	Laboratorial	Clinical	Laboratorial			
Arkhema et al.,^21^ Cohort	*n* = 81,132; 292 with RA	58.5 years	Questionnaire- self-report of periodontal surgery or tooth loss in the last two years	None	Questionnaire self-report of connective tissue disease or had been diagnosed with RA by a physician in the last 2 years	None	Cox regression analysis	RA prognosis for women with periodontal surgery (RR = 1.24; 95% CI 0.83–1.83); RA prognosis for women with tooth loss RR = 1.18; 95% CI (0.47–2.95)	No significant risk of RA for women with periodontal surgery or tooth loss
Chou et al.,^20^ Cohort	*n* = 89,4012: 628.628 with PD group; 168.842 non-PD diagnosis group; 96.542 PD-DS group	PD: 43.9 ± 17.1; DS: 32.1 ± 15.5; PD-DS diagnosis group: 27.9 ± 21.3	ICD9-CM codes 523.3–523.5)	None	At least one ambulatory visit with a diagnosis of RA (ICD9-CM code 714.0)	None	Cox regression analysis; RR and HRs	Risk of RA with PD (HRs 1.89 and 1.43; 95% CI 1.56–2.29) Risk of RA with DS cohort (HRs 1.09 and 1.87; 95% CIs 1.56–2.29) Comparisons of PD cohort and non-PD and DS cohorts; risk of RA (non-PD HR 1.91; 95% CI 1.57–2.30) (DS HR 1.35; 95% CI 1.09–1.67)	Higher risk of RA development for PD cohort and DS cohort (non-PD cohort as reference) PD cohort had a higher risk of RA than the non-PD and PD scale
De Pablo et al.,^22^ CS	*n* = 194:96 PD group:98 healthy	PD: 46 ± 8 years; healthy: 29 ± 7 years.	Clinical and radiographical evaluation	None	Prognosis for RA autoantibodies: CCP, MCV; CEP-1; cit-vim; cit-fib; anti-CParg (negative control of CCP); REP-1; antivimentin and antifibrinogen antibodies	Serum samples of patients with and without PD: ELISA (CCP); MCV; CEP-1; cit-vim; cit-fib; anti-CParg; REP-1; antivimentin and antifibrinogen antibodies	Chi-square, Multiple linear regression: Logistic regression	PD *versus* non-PD: higher frequency of positive anti- CEP-1 (12% *versus* 3%; *p* = 0.02) and its uncitrullinated form anti-REP-1 (16% *versus* 2%; *p* < 0.001); Positive antibodies against uncitrullinated fibrinogen and CParg -PD compared with non-PD (26% *versus* 3%; *p* < 0.001: fibrinogen) (9% *versus* 3%; *p* = 0.06: CParg); PD had 43% (*p* = 0.03), 71% (*p* = 0.002) and 114% (*p* < 0.001) higher anti-CEP-1, anti-REP-1 and antifibrinogen titres, respectively, *versus* non-PD (adjusted); nonsmokers with PD *versus* non-PD: higher titres of anti-CEP-1 (103%, *p* < 0.001), anti-REP-1 (91%, *p* = 0.001), anti-vimentin (87%, *p* = 0.002), and antifibrinogen (124%, *p* < 0.001)	Antibody response in PD is predominantly directed to the uncitrullinated peptides of the RA autoantigens
Demmer et al.,^28^ CS	9702	0–4 MT (*n* = 2029/38 ± 0.3 years); 5–8 MT (*n* = 1990/45 ± 0.3 years); 9–14 MT (*n* = 1559/50 ± 0.3 years; 15–31 MT (*n* = 1814/55 ± 0.3 years); edentulous (*n* = 2310/62 ± 0.3 years)	CPI Russell^29^ Assessment of the presence/absence of periodontal disease for each tooth, tooth mobility, and teeth loss	None	RA definition by self-report of physician diagnosis or physical examination data corresponding to criteria 1–4 of the American Rheumatism Association (1987 criteria)	None	Multivariable logistic regression analysis: hazard ratios (95% CI)	Incident RA ORs in gingivitis 1.32 (0.85–2.06) and PD 1.00 (0.68–1.48) ORs for prevalent RA	Although participants with periodontal disease or ⩾5 MT experienced higher odds of prevalent/incident RA, most ORs were not statistically significant and lacked dose responsiveness Differential RA ascertainment bias complicated the interpretation of these data
Dominguez et al.,^25^ CS	*n* = 280: 80 controls; 80 with RA; 80 with PD; 40 with PD+RA	Control: 48.8 ± 9.86; PD: 45.25 ± 12.0; RA: 47.21 ± 13.3; PD+RA: 48.7 ± 11.5	Probing depth Clinical and CAL	None	Performed by a rheumatologist in accordance with the criteria of the American College of Rheumatology	IL-1α + 4845 (rs17561); IL-1α + 889 (rs1800587); IL-1β + 3954 (rs1143634), IL-1β + 511 (rs16944); TNF-α + 308 (rs1800629)	Multiple linear and logistic regression	No significant association in the genotype frequencies of TNF-α 308 and IL-1α + 4845 SNPs Significant association: IL-1β +511 SNP was positively associated with RA + PD (*p* < 0.0041, OR: 4.84 95% CI: 1.71–13.67) Genotype TT of IL-1β + 3954 SNP positively related (*p* < 0.026, OR: 10.28 95% CI: 1.22–86.64) with PD+RA	Genotypes and Haplotypes of Il-1b are related with PD and RA
Mikuls et al.,^23^ CS	322: Autoantibody negative for RA: 171; autoantibody positive for RA: 113; high risk for PD: 38.	Autoantibody negative for RA: 44 ± 14; autoantibody positive for RA: 48 ± 15; high risk for PD: 51 ± 16.	Self-reported questionnaire about PD signs.	Immunoglobulins of PD-pathogen: anti-*Porphyromonas gingivalis*; anti-*Fusobacterium nucleatum*; Anti-*Prevotella intermedia*	Physician diagnosis or physical (American College of Rheumatology criteria, 1987)	Autoantibodies of RA– ACPA; Rheumatoid factor	Multiple linear regression	Anti-*P. gingivalis*: higherconcentrations on positiveautoantibody groups [ORadj95% (Confidence interval) 1.39 (1.00–1.92) *p* = 0.047] and high-risk group [ORadj 95% (Confidence interval)1.70 (1.05–2.74) *p* = 0.031]	Immunity to *P. gingivalis*, but not *P. intermedia* or *F. nucleatum* is significantly associated with the presence of RA-related autoantibodies in individuals at risk of RA; these results support the hypothesis that infection with *P. gingivalis* may play a central role in the early loss of tolerance to self-antigens that occurs in the pathogenesis of RA
Reichert et al.,^24^ CS	42 patients with RA; Controls: 114	Controls: mean age 53.8 ± 16.7 years; RA: mean age 56.1 ± 15.2 years	API, BOP; MT; CAL	DNA assessment of *Aggregatibacter actinomycetemcomitans; P. gingivalis; Prevotella intermedia; Tannerella forsythia; Treponema denticola* The relationship between the presence of Periodontopathogen DNA on periodontal pockets and synovial fluids of knee joints	The patients with RA were diagnosed by experienced rheumatologists (CS, GK) according to current criteria for classifying RA and spondyloarthropathies	None	Chi-square; Fisher’s exact test, Spearman’s correlation	In patients with RA, DNA of *P. gingivalis* was detected in synovial ﬂuid more often than in controls (15.7% *versus* 3.5%, *p* = 0.045); more patients than controls harbored DNA from *P. gingivalis* in both oral plaque and synovial ﬂuid (11.9% *versus* 0.9%, *p *= 0.030). Among the RA group, the number of MT was correlated with the number of joints with movement restrictions caused by RA, but not significant after Bonferroni’s correction	DNA of periodontopathogens can be found in synovial fluid and oral bacteria may play a role in the pathogenesis of arthritis
Terao et al., 2015^27^ CS	*n* = 9554	53.2 ± 13.43	MT, CPI and CAL	None	Relationship between ACPA positivity and IgM-RF with periodontal status	RF ACPA	Logistic regression	MT–ACPA positivity: OR 95% = 1.03 (1.00–1.05); *p* = 0.024; CPI–ACPA positivity: OR 95% = 1.23 (1.07–1.42); *p* = 0.0042; CAL–ACPA positivity: OR 95% = 1.18 (1.01–1.37); *p* = 0.037; MT–RF positivity: OR 95% = 0.99 (0.98–1.01); *p* = 0.27; CPI–RF positivity: OR 95% = 1.00 (0.92–1.09); *p* = 0.98; CAL–RF positivity: OR 95% = 0.93 (0.84–1.03); *p* = 0.19	The significant associations between PD parameters and positivity and levels of ACPA in healthy population support the major involvement of PD with ACPA production
Thé and Ebersole,^26^ CS	*n* = 260 Controls *n* = 65 Localized juvenile patients *n* = 57 ADP (*n* = 52) Adult PD (*n* = 62)	Normal: Control: 18–53 years; Localized juvenile PD patients: 13–30 years; ADP 12–35 years	Not described	Assessement of PD oral pathogens antibodies: *Actinobacillus actinomyecetemcomitans; Bacteroides denticola; B. gingivalis; B. gracils; B. intermedius; B. melaninogenicus; B. oralis; Campylobacter concisus; Capnocytophaga gingivalis; C. ochracea; C. sputigena*; *Eikenella corrodens; Fusobacterium* *Nucteatum; Wolinelta recta*	Assessment of IgM-RF levels on sera and its association with PD oral pathogens	Assessment of IgM-RF levels on sera and its association with PD oral pathogens	Not described in text	16 of 171 (9.4%) seropositive for IgM-RF The total immunoglobulin levels of the two groups (seropositive and seronegative groups) were not significantly different and the means of both were slightly lower than the RA group (*n* = 10)	The chronic inflammation associated with PD appears to increase the formation of IgM-RF significantly; however, there does appear to be a relationship between IgM-RF and elevated antibody to selected oral micro-organisms

ACPA, antibodies to citrullinated protein antigens; ADP, advanced destructive periodontitis; anti-CParg, (negative control of CCP); API, plaque index; BOP, bleeding on probing; CAL, clinical attachment level/loss; CCP, anticyclic citrullinated peptide; CEP-1, anticitrullinated α-enolase peptide-1; CI, confidence interval; cit-vim, anticitrullinated vimentin, cit-fib, anticitrullinated fibrinogen; CS, cross-sectional; CPI, Community Plaque Index; DNA, deoxyribonucleic acid; DS, dental scaling; HR, hazard ratio; ICD9-CM, International Classification of Diseases, 9th edition, Clinical Modification; IgM, immunoglobulin M; LA, loss of attachment; MCV, antimutated citrullinated vimentin; MT, missing teeth; non-PD, nonperiodontitis; OR, odds ratio; PD, periodontitis; PI, Periodontal Index; RA, rheumatoid arthritis; REP-1, antiarginine-containing α-enolase peptide-1; RF, rheumatoid factor; RR, relative risk.

(1) Associations provided by regression analysis, relative risk, and odds ratio (OR) of periodontitis diagnosis parameters and occurrence of RA;^20^
(2) Associations supplied by laboratory association of periodontitis pathogens and the pro-inflammatory profile of RA;^22–24,26^
(3) Associations provided by periodontitis diagnosis parameters and pro-inflammatory profile of RA.^27^

The periodontal parameters used by the selected studies were tooth loss/missing teeth (MT), probing depth, clinical attachment loss (CAL), bleeding on probing BOP, tooth mobility (TM), radiographic evaluation of periodontitis (PD), recent periodontal surgery, periodontal index (PEI), community periodontal index (CPI), and plaque index (PI). Between those parameters, only four of the selected studies described the evaluated parameters.^21,24,25,28^

Laboratory parameters of periodontitis consist of immunoglobulin pathogens of *P. gingivalis* and deoxyribonucleic acid (DNA) assessment of periodontal pathogens. To correlate pro-inflammatory parameters of RA with periodontitis, the studies evaluate the anticitrullinated autoantibodies (Table 2),^22,23,27^ rheumatoid-factor-immunoglobulin M,^23,27^ and IL gene polymorphisms^25^ related to RA and clinical signs of RA classified by American College of Rheumatologists, 1987.

## Risk of bias

The quality of measurements depicted in the articles is shown in Table 3. Among the nine studies, six were classified as a low risk of bias,^20,22–25,27^ and three as a high risk.^21,26,28^ The high risk of bias was considered due to lack of information regarding the sampling method and statistical analysis in one study.^26^ The other two articles with an increased risk of bias reported a poor periodontal disease, which may result in outcomes possibly converging to a specific direction.^21,28^

**Table 3.** Quality assessment of studies included, according to Fowkes and Fulton.^13^

	Checklist	Arkema et al.^21^	Chou et al.^20^	De Pablo et al.^22^	Demmer et al.^28^	Dominguez et al.^25^	Mikuls et al.^23^	Reichert et al.^24^	Terao et al.^27^	Thé et al.^26^
Study design appropriate to objectives?	Objective common design									
	Prevalence cross-sectional									
	Prognosis cohort	0	0		0		0			
	Treatment-controlled trial									
	Cause cohort, case control, cross-sectional			0		0		0	0	0
Study sample representative?	Source of sample	0	0	0	0	0	0	0	0	+
	Sampling method	0	0	++	0	++	0	++	+	++
	Sample size	0	0	+	0	+	0	+	0	++
	Entry criteria/exclusion	+	+	+	+	+	+	0	0	0
	Nonrespondents	0	0	0	0	0	0	0	0	0
Control group acceptable?	Definition of controls	0	0	NA	NA	0	NA	0	NA	+
	Source of controls	0	0	NA	NA	+	NA	+	NA	0
	Matching/randomization	NA	+	NA	NA	+	NA	0	NA	0
	Comparable characteristics	0	0	0	0	0	0	+	0	0
Quality of measurements and outcomes?	Validity	++	0	0	++	0	0	0	0	+
	Reproducibility	0	0	0	0	0	0	+	0	+
	Blindness	0	0	0	0	NA	0	0	0	0
	Quality control	+	0	+	0	0	0	0	0	+
Completeness	Compliance	0	0	0	0	0	0	0	0	0
	Dropouts	0		0	0	0	0	0	0	0
	Deaths	NA	NA	NA	NA	0	NA	NA	NA	0
	Missing data	0	0	0	0	0	0	0	0	0
Distorting influences?	Extraneous treatments	0	0	0	0	0	+	0	0	0
	Contamination	+	0	0	0	0	0	0	0	0
	Changes over time	0	0	0	0	0	0	+	+	0
	Confounding factors	++	+	0	++	+	+	0	0	0
	Distortion reduced by analysis	0	0	NA	NA	0	0	NA	0	NA
	Bias: are the results erroneously biased in a certain direction?	Yes	No	No	Yes	No	No	No	No	Yes
	Confounding: are there any serious confusing or other distorting influences?	Yes	No	No	Yes	No	No	No	No	Yes
	Chance: is it likely that the results occurred by chance?	No	No	No	No	No	No	No	No	Yes

0, no problem; +, minor problem; ++, major problem; NA, not applicable due to type of study.

## Qualitative synthesis of studies

Five of nine studies evaluated subsamples of national cohorts.^20,21,23,27,28^ The countries approached in these studies were Sweden,^21^ Denmark,^28^ Japan,^27^ Taiwan,^20^ and the United States.^23^ Some studies evaluated the clinical parameters of periodontitis (CAL, BOP, etc.) only for diagnosis, but description of values in text was absent. The other studies evaluated samples using biochemical analysis of rheumatoid factors,^22,24,26^ and genetic polymorphisms of cytokines associated with RA.^25^

Most problems relating to the articles were lack of sampling methods,^22,24–26^ the entry criteria/exclusion (participants with smoking habits or systemic diseases),^20–23,25,28^ and small sample size.^22–26^

The association of periodontitis and RA was found in seven of nine articles.^20,22–27^ The link was established by comparison of levels of antibodies to citrullinated protein antigens/protein peptides (ACPA)/rheumatoid factor between groups,^22–24,26,27^ the presence of bacterial DNA on synovial joints,^24,26^ and the association of the prevalence of two diseases after regression analysis of public health data.^20^

## Quantitative analysis of studies

Five studies presented their results only in graphics,^22–24,26,27^ and one study did not provide the total number of patients in case and control groups.^21^ Unfortunately, these authors have not returned the contact attempts and were excluded from the meta-analysis. Only three studies were included in quantitative synthesis.^20,25,28^ Two of these studies were classified as having a low risk of bias,^20,25^ and one as a high risk of bias.^28^

**Figure 2.**
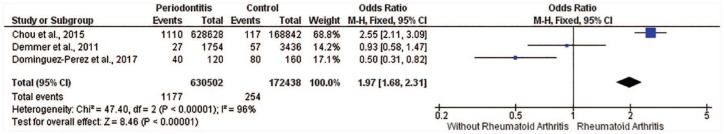
Forest plot of meta-analysis for three studies (*I*^2^ = 96%). The association between periodontitis and RA. CI, confidence interval; M-H, Mantel-Haenszel method; RA, rheumatoid arthritis.

The first meta-analysis of the three studies,^20,25,28^ indicated a considerable heterogeneity (*I*^2^ = 96%, *p* < 0.00001, Figure 2). The periodontitis group had 630,502 participants, and the control group (without periodontitis) had 172,438 participants. The results showed greater RA prevalence for periodontitis (*n* = 1177) than controls (*n* = 254), demonstrating positive association (*p* < 0.00001) between them (OR 1.97; CI 1.68–2.31).

In an attempt to reduce heterogeneity, a sensitivity analysis was performed. Removing studies, one by one, the heterogeneity ranges from 97% to 69%. Therefore, the study of Chou *et al*.^20^ was excluded, and the *I*^2^ = 69% was considered.

**Figure 3.**
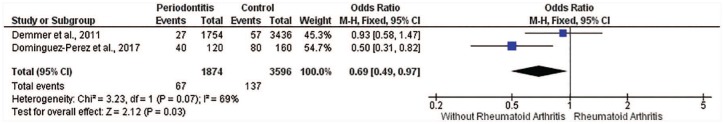
Forest plot of meta-analysis for two studies (*I*^2^ = 69%). The association between periodontitis and RA. CI, confidence interval; M-H, Mantel-Haenszel method; RA, rheumatoid arthritis.

The second meta-analysis shows two studies.^25,28^ The periodontitis group had 1874 participants, and the control group had 3596 participants. A result contrary to the first meta-analysis was observed. The results showed lower RA prevalence for person with periodontitis (*n* = 67) than for controls (*n* = 137), demonstrating negative association (*p* = 0.03) between these variables (OR 0.69; CI 0.49–0.97; Figure 3).

## Discussion

The objective of this review was to identify risk factors related to the association of periodontitis exposure and RA. The qualitative synthesis of nine studies indicates relationships among the diseases (seven of nine studies), but the methodological heterogeneity of our meta-analysis means there is inconclusivity about the association of RA and periodontitis.

Systematic reviews can synthesize and critically evaluate the primary outcomes of investigations through specific strategies to limit bias and random errors. Thus, those reviews can produce supporting information for clinical decisions based on research evidence. In addition to defining key interventions, systematic reviews can also demonstrate ways to achieve decision making where knowledge is lacking.^30^

According to the epidemiological aspects of the included studies, four of the chosen studies showed a comparable prevalence of RA in patients with periodontitis. About 3% incidence and a proportion of 3:1 women:men cases were shown.^31^ Periodontitis cases have shown an elevated ratio/relative risk for RA development, as demonstrated in other studies.^32^

However, some of the selected studies found no correlation between periodontitis parameters and the occurrence of RA.^21,28^ The evaluation parameters are related to tooth loss, the number of teeth, and recent periodontal surgery. It is well known that periodontitis diagnosis is a criterion correlated with related symptoms (gingival bleeding, halitosis, TM, others), natural heritage, medical history, and clinical signs (gingival recession, root exposure, TM, periodontal pockets with bleeding).^3,4^ Tooth loss is one of the measurements that demonstrated sensitivity to show a periodontal compromising of dental tissue.^33^ Nevertheless, tooth loss can be associated with dental caries and other diseases. So, associations of tooth loss may generate false-negative errors, mainly when previous infections of periodontal tissue is not present in patients.^28^

Although, other studies promote synthesis of many related signs of periodontitis using few indices. Hence, the gold standard index, CAL,^1,34,35^ in a full mouth examination is difficult to execute, time consuming, and unfeasible in larger-sample studies.^36^ Therefore, analyzing indices with low specificity may generate misclassifications and weak associations between periodontitis and arthritis.^37^

Among the six articles classified as having low risk of bias, the critical aspects to qualify with low risk of bias were: (a) periodontal characterization of the patient’s condition; periodontal conditions are mainly verified through clinical examination, being CAL, BOP, and PD, the most sensitive indices to periodontal evaluation;^4,38^ and (b) reduction of distortion influences. Matching/randomization and statistical reduction of distortions are essential tools that allow the assessment of study groups with equal probabilities.^39^ Mathematical models that simplify the analysis only for chosen evaluated outcomes may reduce other influences like personal habits, systemic diseases, and environmental impacts.

Our meta-analysis shows an important heterogeneity (*I*^2^ = 96%, *p* < 0.00001). One of the articles^20^ significantly demonstrates the association of periodontitis with RA development. The sensitivity analysis changed the results of the association between periodontitis and RA in the meta-analysis. This suggests that methodological inconsistencies (as suggested by Demmer *et al*.^28^) regarding definition of periodontitis, and fewer proportions of RA/periodontitis patients analyzed (as suggested by Dominguez *et al*.^25^), are still present among the studies included in this meta-analysis.

In our analysis, the misclassification of both diseases may lead to errors in outcomes. Therefore, more methodologically similar studies with numerical data and full periodontal analysis are necessary to clarify the real association between these two factors.

Analyzing the topics presented by articles with a positive association, IL-1β and TNF-α were responsible for many immune signaling pathways and protection of infection injuries.^40^ Some of these functions are related to the bone remodeling pathway through combination with functions of receptor activator of nuclear-kappa-B ligand and its receptors. In periodontitis, TNF-α and IL-1β are actively present and are secreted by monocytes and macrophages, resulting in effects on the bone remodeling pathway. In cases of inflammatory response against bacterial infection, imbalance of bone remodeling may occur, resulting in the alveolar bone loss.^41^

In RA, a systemic increase of TNF-α and IL-1β on gingival crevicular fluid and injured joints was reported, as well as an increase in the periodontal pathogens.^40–42^ Since then, theories about the developing relationship between RA and periodontitis have been discussed; mainly the two-hit model (periodontitis as a first hit, occasioning inflammation, leading to RA, the second hit).^9^ The articles assessed in this review show a critical relationship relating to shared genetic risks involving the cytokine polymorphisms and the human leukocytes antigen–DRB1 allele^23,28^ as well as the activity of periodontal pathogens.

The HLA-DRB1 allele is responsible for 25–50% of vulnerability to RA and, more recently, authors identified this allele as a mass producer of cyclic citrullinated autoantibody peptides (anti-CCP).^43^ These macromolecules are the first epitopes that provoke autoimmune activity by macrophages on joints.^44^ The possible association between periodontitis and RA converges to the central hypothesis of the relationship, in which the citrullinated antigens can cause changes in synovial joints.^45^

Recent studies have shown the presence of periodontal bacteria DNA in synovial joints of patients with established RA.^8,43^ Moreover, further authors have associated the formation of immune complexes in synovial joints involving the *P. gingivalis*, and recently, the *A. actinomycetecomitans*,^8^ the leading Gram-negative periodontal pathogens.

As reported in one of the included studies,^23^ cases of periodontitis and presence of HLA-DRB1 expression result in aggravation and a possible development of RA. So, the treatment of RA, a complex investigation of symptomatology,^46^ requires another health concern regarding oral pathology aspects. If the two-hit model theory becomes a plausible mechanism of RA development in cases of periodontitis (more studies needed), we suggest a combined treatment involving a careful periodontal evaluation considering different measures (health promotion, health prevention, and surgery interventions) to reduce the clinical signs and symptomatology of periodontitis.^46^ Hence, the chance of RA development may be reduced, and the comorbidity in establishing cases of RA can be decreased as well.

The limitations of our study were the absence of research data that share methodological parameters to depict associations between periodontitis and RA. Among the selected articles, two articles that did not find associations directed our finding to inconclusivity regarding our review objective.

## Conclusion

The evidence from the included articles suggests a link between RA and periodontitis genetic risks, bacterial infection, and the typical pro-inflammatory profile shared between RA and periodontitis are key to possible RA development. Our meta-analysis, due to high heterogeneity, showed inconclusive results in the association between these two pathologies. So, more studies with representative samples and defined periodontal evaluation are necessary to establish this possible association of clinical relevance of periodontal treatment in prevention of RA.

## Supplemental Material

Supplementary_file_1_Table – Supplemental material for Does periodontitis represent a risk factor for rheumatoid arthritis? A systematic review and meta-analysis Click here for additional data file. Supplemental material, Supplementary_file_1_Table for Does periodontitis represent a risk factor for rheumatoid arthritis? A systematic review and meta-analysis by Railson de Oliveira Ferreira, Raíra de Brito Silva, Marcela Baraúna Magno, Anna Paula Costa Ponte Sousa Carvalho Almeida, Nathália Carolina Fernandes Fagundes, Lucianne Cople Maia and Rafael Rodrigues Lima in Therapeutic Advances in Musculoskeletal Disease

## Supplemental Material

Supplementary_file_2_Table – Supplemental material for Does periodontitis represent a risk factor for rheumatoid arthritis? A systematic review and meta-analysis Click here for additional data file. Supplemental material, Supplementary_file_2_Table for Does periodontitis represent a risk factor for rheumatoid arthritis? A systematic review and meta-analysis by Railson de Oliveira Ferreira, Raíra de Brito Silva, Marcela Baraúna Magno, Anna Paula Costa Ponte Sousa Carvalho Almeida, Nathália Carolina Fernandes Fagundes, Lucianne Cople Maia and Rafael Rodrigues Lima in Therapeutic Advances in Musculoskeletal Disease

## Supplemental Material

Supplementary_file_3_Table – Supplemental material for Does periodontitis represent a risk factor for rheumatoid arthritis? A systematic review and meta-analysis Click here for additional data file. Supplemental material, Supplementary_file_3_Table for Does periodontitis represent a risk factor for rheumatoid arthritis? A systematic review and meta-analysis by Railson de Oliveira Ferreira, Raíra de Brito Silva, Marcela Baraúna Magno, Anna Paula Costa Ponte Sousa Carvalho Almeida, Nathália Carolina Fernandes Fagundes, Lucianne Cople Maia and Rafael Rodrigues Lima in Therapeutic Advances in Musculoskeletal Disease
