# Supplementary material for: Does periodontitis represent a risk factor for rheumatoid arthritis? A systematic review and meta-analysis
Source: Ther Adv Musculoskelet Dis. 2019 Jul 9;11:1759720X19858514. doi: 10.1177/1759720X19858514 (PMC6620730; doi:10.1177/1759720X19858514)
Supplement: Supplementary_file_2_Table – Supplemental material for Does periodontitis represent a risk factor for rheumatoid arthritis? A systematic review and meta-analysis [file Supplementary_file_2_Table.docx]

**S2 Table: Terms used on database search.**

| Database | Search format |
| --- | --- |
| PUBMED | ((((((((humans[MeSH Terms]) OR Man, modern[Title/Abstract]) OR Modern Man[Title/Abstract]) OR Man taxonomy[Title/Abstract]) OR Homo sapiens[Title/Abstract]) OR human[Title/Abstract])) AND ((((((((((((rheumatoid factor[MeSH Terms]) OR arthritis, rheumatoid[MeSH Terms]) OR joints[MeSH Terms]) OR joint diseases[MeSH Terms]) OR factor, rhematoid[Title/Abstract]) OR rheumatoid arthritis[Title/Abstract]) OR joint diseases[Title/Abstract]) OR joint[Title/Abstract]) OR fluid, synovial[Title/Abstract]) OR fluids, synovial[Title/Abstract]) OR synovial fluids[Title/Abstract]) OR synovia[Title/Abstract])) AND (((((((((((((((((((((((((((((((((((((((((((((((periodontitis[MeSH Terms]) OR periodontal diseases[MeSH Terms]) OR periodontal atrophy[MeSH Terms]) OR alveolar bone loss[MeSH Terms]) OR chronic periodontitis[MeSH Terms]) OR periodontitides[Title/Abstract]) OR pericementitis[Title/Abstract]) OR pericementitides[Title/Abstract]) OR disease, periodontal[Title/Abstract]) OR diseases, periodontal[Title/Abstract]) OR periodontal diseases[Title/Abstract]) OR parodontosis[Title/Abstract]) OR parodontoses[Title/Abstract]) OR pyorrhea alveolaris[Title/Abstract]) OR periodontal atrophies[Title/Abstract]) OR atrophy of periodontium[Title/Abstract]) OR periodontium atrophies[Title/Abstract]) OR periodontium atrophy[Title/Abstract]) OR gingivo-osseus atrophy[Title/Abstract]) OR gingivo osseus atrophy[Title/Abstract]) OR gingivo-osseus atrophies[Title/Abstract]) OR alveolar bone losses[Title/Abstract]) OR alveolar process atrophy[Title/Abstract]) OR alveolar process atrophies[Title/Abstract]) OR alveolar resorption[Title/Abstract]) OR alveolar resorptions[Title/Abstract]) OR resorption, alveolar[Title/Abstract]) OR resorptions, alveolar[Title/Abstract]) OR bone loss, periodontal[Title/Abstract]) OR bone losses, periodontal[Title/Abstract]) OR periodontal bone losses[Title/Abstract]) OR periodontal bone loss[Title/Abstract]) OR periodontal resorption[Title/Abstract]) OR periodontal resorptions[Title/Abstract]) OR resorption, periodontal[Title/Abstract]) OR alveolar bone atrophy[Title/Abstract]) OR alveolar bone atrophies[Title/Abstract]) OR bone atrophies, alveolar[Title/Abstract]) OR bone atrophy, alveolar[Title/Abstract]) OR bone loss, alveolar[Title/Abstract]) OR chronic periodontitides[Title/Abstract]) OR periodontitides, chronic[Title/Abstract]) OR periodontitis, chronic[Title/Abstract]) OR adult periodontitis[Title/Abstract]) OR adult periodontitides[Title/Abstract]) OR periodontitides, adults[Title/Abstract]) OR periodontitis adult[Title/Abstract]) |
| SCOPUS | Humans or "Man, modern" or "Modern man" or "Man taxonomy" or "Homo sapiens" or Human AND "Rheumatoid factor" or "Arthritis, Rheumatoid" or Joints or "Joint Diseases" or "Factor, Rheumatoid" or "Rheumatoid Arthritis" or joint or "Joint disease" or "Fluid, Synovial" or "Fluids, Synovial" or "Synovial Fluids" or Synovia AND Periodontitis or Periodontitides or Pericementitis or Pericementitides or "Periodontal Diseases" or "Disease, Periodontal" or "Diseases, Periodontal" or "Periodontal Disease" or Parodontosis or Parodontoses or "Pyorrhea Alveolaris" or "Periodontal Atrophy" or "Periodontal Atrophies" or "Atrophy of Periodontium" or "Periodontium Atrophies" or "Periodontium Atrophy" or "Gingivo-Osseous Atrophy" or "Gingivo Osseous Atrophy" or "Gingivo-Osseous Atrophies" or "Alveolar Bone Loss" or "Alveolar Bone Losses" or "Alveolar Process Atrophy" or "Alveolar Process Atrophies" or "Alveolar Resorption" or "Alveolar Resorptions" or "Resorption, Alveolar" or "Resorptions, Alveolar" or "Bone Loss, Periodontal" or "Bone Losses, Periodontal" or "Periodontal Bone Losses" or "Periodontal Bone Loss" or "Periodontal Resorption" or "Periodontal Resorptions" or "Resorption, Periodontal" or "Alveolar Bone Atrophy" or "Alveolar Bone Atrophies" or "Bone Atrophies, Alveolar" or "Bone Atrophy, Alveolar" or "Bone Loss, Alveolar" or "Chronic Periodontitis" or "Chronic Periodontitides" or "Periodontitides, Chronic" or "Periodontitis, Chronic" or "Adult Periodontitis" or "Adult Periodontitides" or "Periodontitides, Adult" or "Periodontitis, Adult" |
| COCHRANE | Humans or "Man, modern" or "Modern man" or "Man taxonomy" or "Homo sapiens" or Human AND "Rheumatoid factor" or "Arthritis, Rheumatoid" or Joints or "Joint Diseases" or "Factor, Rheumatoid" or "Rheumatoid Arthritis" or joint or "Joint disease" or "Fluid, Synovial" or "Fluids, Synovial" or "Synovial Fluids" or Synovia AND Periodontitis or Periodontitides or Pericementitis or Pericementitides or "Periodontal Diseases" or "Disease, Periodontal" or "Diseases, Periodontal" or "Periodontal Disease" or Parodontosis or Parodontoses or "Pyorrhea Alveolaris" or "Periodontal Atrophy" or "Periodontal Atrophies" or "Atrophy of Periodontium" or "Periodontium Atrophies" or "Periodontium Atrophy" or "Gingivo-Osseous Atrophy" or "Gingivo Osseous Atrophy" or "Gingivo-Osseous Atrophies" or "Alveolar Bone Loss" or "Alveolar Bone Losses" or "Alveolar Process Atrophy" or "Alveolar Process Atrophies" or "Alveolar Resorption" or "Alveolar Resorptions" or "Resorption, Alveolar" or "Resorptions, Alveolar" or "Bone Loss, Periodontal" or "Bone Losses, Periodontal" or "Periodontal Bone Losses" or "Periodontal Bone Loss" or "Periodontal Resorption" or "Periodontal Resorptions" or "Resorption, Periodontal" or "Alveolar Bone Atrophy" or "Alveolar Bone Atrophies" or "Bone Atrophies, Alveolar" or "Bone Atrophy, Alveolar" or "Bone Loss, Alveolar" or "Chronic Periodontitis" or "Chronic Periodontitides" or "Periodontitides, Chronic" or "Periodontitis, Chronic" or "Adult Periodontitis" or "Adult Periodontitides" or "Periodontitides, Adult" or "Periodontitis, Adult" in Title, Abstract, Keywords |
| WEB OF SCIENCE | TS=(Humans OR "Man, modern" OR "Modern man" OR "Man taxonomy" OR "Homo sapiens" OR Human) AND TS=("Rheumatoid factor" OR "Arthritis, Rheumatoid" OR Joints OR "Joint Diseases" OR "Factor, Rheumatoid" OR "Rheumatoid Arthritis" OR joint OR "Joint disease" OR "Fluid, Synovial" OR "Fluids, Synovial" OR "Synovial Fluids" OR Synovia) AND TS=(Periodontitis OR Periodontitides OR Pericementitis OR Pericementitides OR "Periodontal Diseases" OR "Disease, Periodontal" OR "Diseases, Periodontal" OR "Periodontal Disease" OR Parodontosis OR Parodontoses OR "Pyorrhea Alveolaris" OR "Periodontal Atrophy" OR "Periodontal Atrophies" OR "Atrophy of Periodontium" OR "Periodontium Atrophies" OR "Periodontium Atrophy" OR "Gingivo-Osseous Atrophy" OR "Gingivo Osseous Atrophy" OR "Gingivo-Osseous Atrophies" OR "Alveolar Bone Loss" OR "Alveolar Bone Losses" OR "Alveolar Process Atrophy" OR "Alveolar Process Atrophies" OR "Alveolar Resorption" OR "Alveolar Resorptions" OR "Resorption, Alveolar" OR "Resorptions, Alveolar" OR "Bone Loss, Periodontal" OR "Bone Losses, Periodontal" OR "Periodontal Bone Losses" OR "Periodontal Bone Loss" OR "Periodontal Resorption" OR "Periodontal Resorptions" OR "Resorption, Periodontal" OR "Alveolar Bone Atrophy" OR "Alveolar Bone Atrophies" OR "Bone Atrophies, Alveolar" OR "Bone Atrophy, Alveolar" OR "Bone Loss, Alveolar" OR "Chronic Periodontitis" OR "Chronic Periodontitides" OR "Periodontitides, Chronic" OR "Periodontitis, Chronic" OR "Adult Periodontitis" OR "Adult Periodontitides" OR "Periodontitides, Adult" OR "Periodontitis, Adult") |
| OPENGREY | "Chronic Periodontitis" AND "Eheumatoid Arthritis" |
| LILACS | (tw:(Humans OR "Man, modern" OR "Man (taxonomy)" OR "Modern man" OR "Homo sapiens" OR Human)) AND (tw:"Rheumatoid Factor" OR "Arthritis, Rheumatoid" OR Joints OR "Joint diseases" OR "Factor, Rheumatoid" OR "Rheumatoid Arthritis" OR Joint OR "Joint Disease" OR "Fluid, Synovial" OR "Fluids, Synovial" OR "Synovial Fluids" OR Synovia)) AND (tw:(Periodontitis OR Periodontitides OR Pericementitis OR Pericementitides OR "Periodontal Diseases" OR "Disease, Periodontal" OR "Diseases, Periodontal" OR "Periodontal Disease" OR Parodontosis OR Parodontoses OR "Pyorrhea Alveolaris" OR "Periodontal Atrophy" OR "Periodontal Atrophies" OR "Atrophy of Periodontium" OR "Periodontium Atrophies" OR "Periodontium Atrophy" OR "Gingivo-Osseous Atrophy" OR "Gingivo Osseous Atrophy" OR "Gingivo-Osseous Atrophies" OR "Alveolar Bone Loss" OR "Alveolar Bone Losses" OR "Alveolar Process Atrophy" OR "Alveolar Process Atrophies" OR "Alveolar Resorption" OR "Alveolar Resorptions" OR "Resorption, Alveolar" OR "Resorptions, Alveolar" OR "Bone Loss, Periodontal" OR "Bone Losses, Periodontal" OR "Periodontal Bone Losses" OR "Periodontal Bone Loss OR "Periodontal Resorptions OR "Periodontal Resorption" OR "Resorption, Periodontal" OR "Alveolar Bone Atrophy" OR "Alveolar Bone Atrophies" OR "Bone Atrophies, Alveolar" OR "Bone Atrophy, Alveolar" OR "Bone Loss, Alveolar" OR "Chronic Periodontitis" OR "Chronic Periodontitis" OR "Periodontitides, Chronic" OR "Periodontitis, Chronic" OR "Adult Periodontitis" OR "Adult Periodontitides" OR "Periodontitides, Adult" OR "Periodontitis, Adult")) |
| GOOGLE SCHOLAR | "Chronic periodontitis" + “Rheumatoid Arthritis - "in vitro" – “systematic review” -book |
| CLINICAL TRIALS.GOV | Periodontitis AND “Rheumatoid Arthritis” |
