# Supplementary material for: Does periodontitis represent a risk factor for rheumatoid arthritis? A systematic review and meta-analysis
Source: Ther Adv Musculoskelet Dis. 2019 Jul 9;11:1759720X19858514. doi: 10.1177/1759720X19858514 (PMC6620730; doi:10.1177/1759720X19858514)
Supplement: Supplementary_file_3_Table – Supplemental material for Does periodontitis represent a risk factor for rheumatoid arthritis? A systematic review and meta-analysis [file Supplementary_file_3_Table.docx]

**S3 Table: Articles excluded after full-text analysis and reasons for exclusions**

| Reference | Reason for exclusion |
| --- | --- |
| 1. Ishida K1, Kobayashi T, Ito S, Komatsu Y, Yokoyama T, Okada M, Abe A, Murasawa A, Yoshie H. Interleukin-6 gene promoter methylation in rheumatoid arthritis and chronic periodontitis. J Periodontol. 2012 Jul;83(7):917-25. doi: 10.1902/jop.2011.110356. Epub 2011 Nov 28 | Establish cases of RA |
| 1. . Gargiulo AV Jr, Robinson J, Toto PD, Gargiulo AW.Identification of rheumatoid factor in periodontal disease. J Periodontol. 1982 Sep;53(9):568-77. | Establish cases of RA |
| 1. Chen HH, Huang N, Chen YM, Chen TJ, Chou P, Lee YL, et al. Association between a history of periodontitis and the risk of rheumatoid arthritis: a nationwide, population-based, case-control study. Annals of the rheumatic diseases. 2013;72(7):1206-11 | Establish cases of RA |
| 1. Konig MF, Abusleme L, Reinholdt J, et al. Aggregatibacter actinomycetemcomitans-induced hypercitrullination links periodontal infection to autoimmunity in rheumatoid arthritis. Science translational medicine. 2016;8(369):369ra176. doi:10.1126/scitranslmed.aaj1921. | Establish cases of RA |
| 1. Hashimoto M, Yamazaki T, Hamaguchi M, et al. Periodontitis and Porphyromonas gingivalis in Preclinical Stage of Arthritis Patients. Heymann D, ed. PLoS ONE. 2015;10(4):e0122121. doi:10.1371/journal.pone.0122121. | Establish cases of RA symptomatology |
